# Acquired Concurrent EGFR T790M and Driver Gene Resistance From EGFR-TKIs Hampered Osimertinib Efficacy in Advanced Lung Adenocarcinoma: Case Reports

**DOI:** 10.3389/fphar.2022.838247

**Published:** 2022-04-06

**Authors:** Yue Zeng, Yuanqing Feng, Guihua Fu, Junlan Jiang, Xiaohan Liu, Yue Pan, Chunhong Hu, Xianling Liu, Fang Wu

**Affiliations:** ^1^ Department of Oncology, The Second Xiangya Hospital, Central South University, Changsha, China; ^2^ Department of Oncology, Xiangtan Central Hospital, Xiangtan, China; ^3^ Jiangnan Hospital, Xiangtan, China; ^4^ Hunan Cancer Mega-Data Intelligent Application and Engineering Research Centre, Changsha, China; ^5^ Hunan Key Laboratory of Tumor Models and Individualized Medicine, The Second Xiangya Hospital, Central South University, Changsha, China; ^6^ Hunan Key Laboratory of Early Diagnosis and Precision Therapy in Lung Cancer, The Second Xiangya Hospital, Central South University, Changsha, China

**Keywords:** non-small-cell lung cancer, EGFR-TKI acquired resistance, T790M, diver gene resistance, ALK fusion, MET amplification, RET fusion

## Abstract

The acquired resistance of epidermal growth factor receptor tyrosine kinase inhibitors (EGFR-TKIs) is inevitable and heterogeneous. The strategies to overcome acquired resistance are significant. For patients with secondary T790M-positive after early generation EGFR-TKIs, osimertinib is the standard second-line therapy. In patients resistant to prior early generation EGFR-TKIs, the acquired T790M mutation overlaps with other driver gene resistance, such as HER2-and MET amplification, accounting for 4–8%. The efficacy of osimertinib is unclear in patients with concurrent multiple driver gene resistance. We here report a patient who acquired EGFR T790M, STRN-ALK fusion, and EGFR amplification after gefitinib progression and subsequent MET amplification acquired from osimertinib. The other patient acquired EGFR T790M and MET amplification post-dacomitinib and acquired CCDC6-RET fusion after osimertinib treatment. Besides, subsequent new bypass activations were the possible resistance mechanisms to second-line osimertinib. Both patients had progression-free survival (PFS) less than 4 months and limited benefits from osimertinib second-line therapy. The T790M accompanying driver gene resistance will be a new subtype after EGFR-TKIs progression, needing effective treatment options.

## Introduction

The resistance mechanisms to epidermal growth factor receptor tyrosine kinase inhibitors (EGFR-TKIs) are inevitable and heterogeneous, which restricts the clinical benefits (Piper-Vallillo et al., 2020). The most frequent acquired resistance mechanism of early generation EGFR-TKIs is secondary T790M mutation, and osimertinib is the standard second-line therapy (Mok et al., 2017; Westover et al., 2018). Some oncogene alterations like ALK, ROS1, and RET are increasingly observed in resistance mechanisms (Piper-Vallillo et al., 2020). Concurrent driver gene alteration as a resistance mechanism occurs in lung adenocarcinoma with a low incidence rate. However, no good consensus was formulated when EGFR T790M mutation was combined with other driver gene resistance. Herein, we reported two cases that developed T790M-positive and driver gene resistance after receiving EGFR-TKIs, which provide valuable suggestions for clinical decisions. Further, extensive literature reviews were made to summarize the reported cases with T790M mutation and driver gene resistance in NSCLC patients and to analyze the prognosis and efficacy of osimertinib.

## Case Report

### # Case 1

**FIGURE 1:**
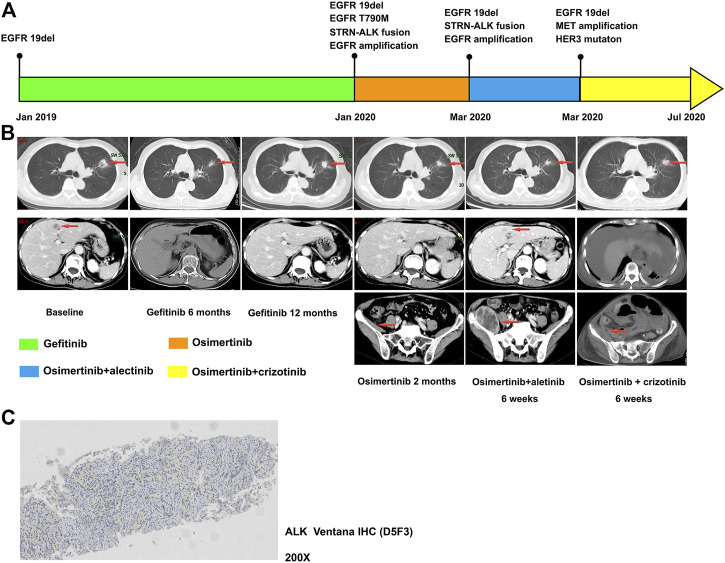
**(A)**: Timer shaft of gene testing and treatment measurement of case 1; **(B)**: Computed tomographic images of primary lung lesion, liver, and iliopsoas mass metastases; **(C)**: ALK Ventana immunohistochemistry (D5F3) as negative.

Case 1 reports a 75-year-old non-smoking woman suffering from cough and pain in her upper limbs and back. Computed tomography (CT) identified a nodule in the left superior lung and metastasis in the liver in October 2018 (Figure 1B). Single-photon emission computed tomography bone imaging showed multiple bone metastases. The pathology was confirmed as lung adenocarcinoma and ALK Ventana immunohistochemistry (D5F3) was negative (Figure 1C). Besides, genotype by amplification-refractory mutation system polymerase chain reaction (RT-PCR) revealed an EGFR 19 exon in-frame deletion (19del) (Supplementary Table S1). She started on gefitinib at 250 mg daily in December 2018 (Figure 1A). Partial response was obtained in the patient with liver metastasis disappearing. The disease progressed with lung lesion enlargement and bone metastases after 12 months. Plasma-based next-generation sequencing (NGS, 168-gene panel, Burning Rock, Guangzhou) in December 2019 showed an EGFR 19del, EGFR T790M, STRN-ALK fusion, TP53 mutation, and EGFR amplification. Then she began osimertinib at 80 mg daily, which controlled her lung mass within 2 months. Alectinib was combined with osimertinib since plasma-based NGS showed that the STRN-ALK frequency was increasing. The STRN-ALK frequency disappeared after 3 weeks of treatment. However, new liver and right iliopsoas metastases were identified after 7 weeks of combination treatment. The CT-guided needle biopsy of the right iliopsoas lesion revealed metastatic lung adenocarcinoma, and tissue-based NGS showed an EGFR 19del, MET amplification, and HER3 mutation (425 gene-panel, Geneseeq Technology Inc., Nanjing). Crizotinib combined with osimertinib was administrated. Due to gastrointestinal toxicity, crizotinib was reduced to 250 mg daily. The patient showed remission of the right iliopsoas mass after 2 months of treatment, but her clinical conditions worsened, and she eventually died 1 week later.

### # Case 2

**FIGURE 2:**
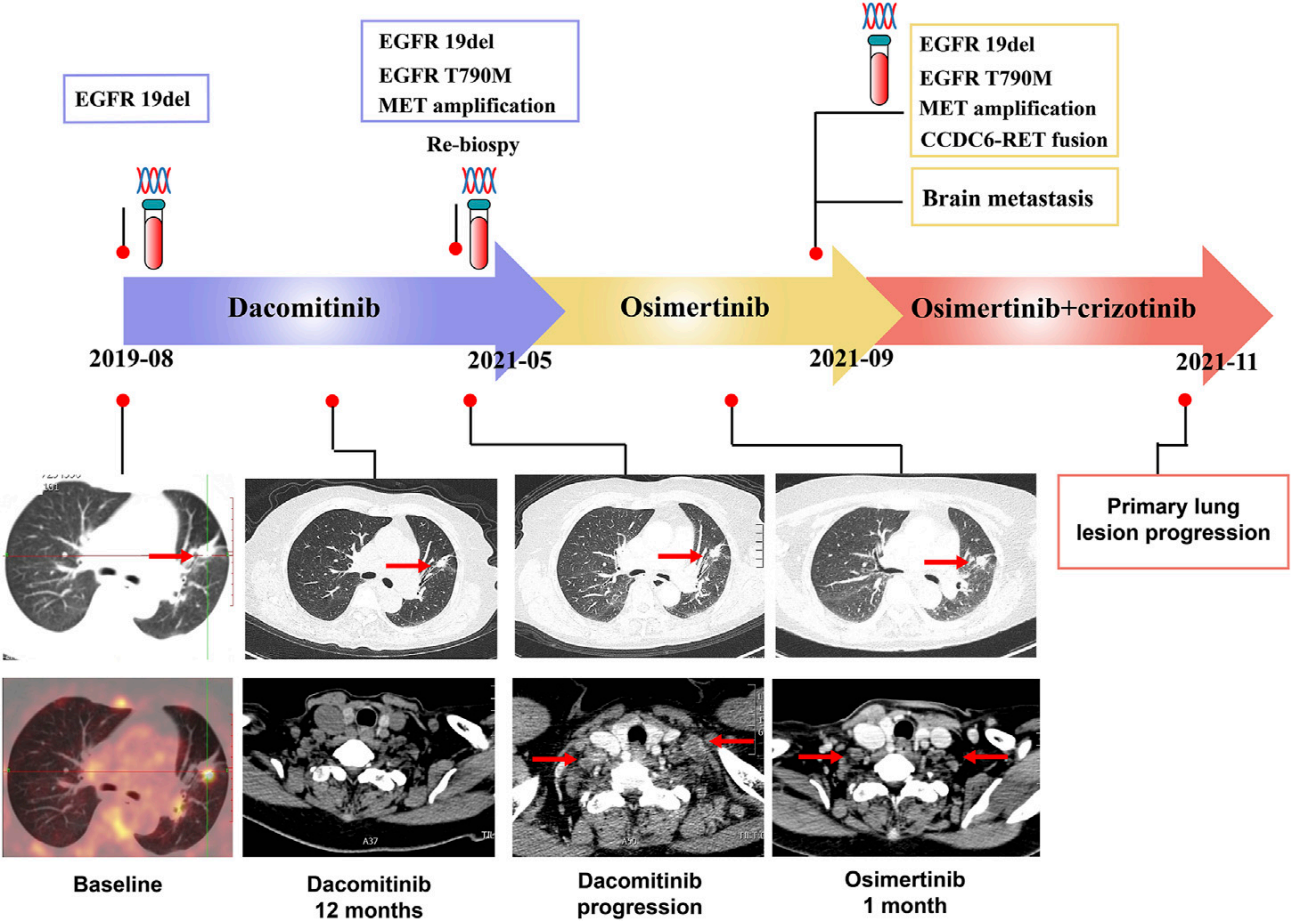
Timer shaft of gene testing and treatments measurement, and the computed tomographic images of primary lung lesion and lymph nodes metastases of case 2.

**FIGURE 3:**
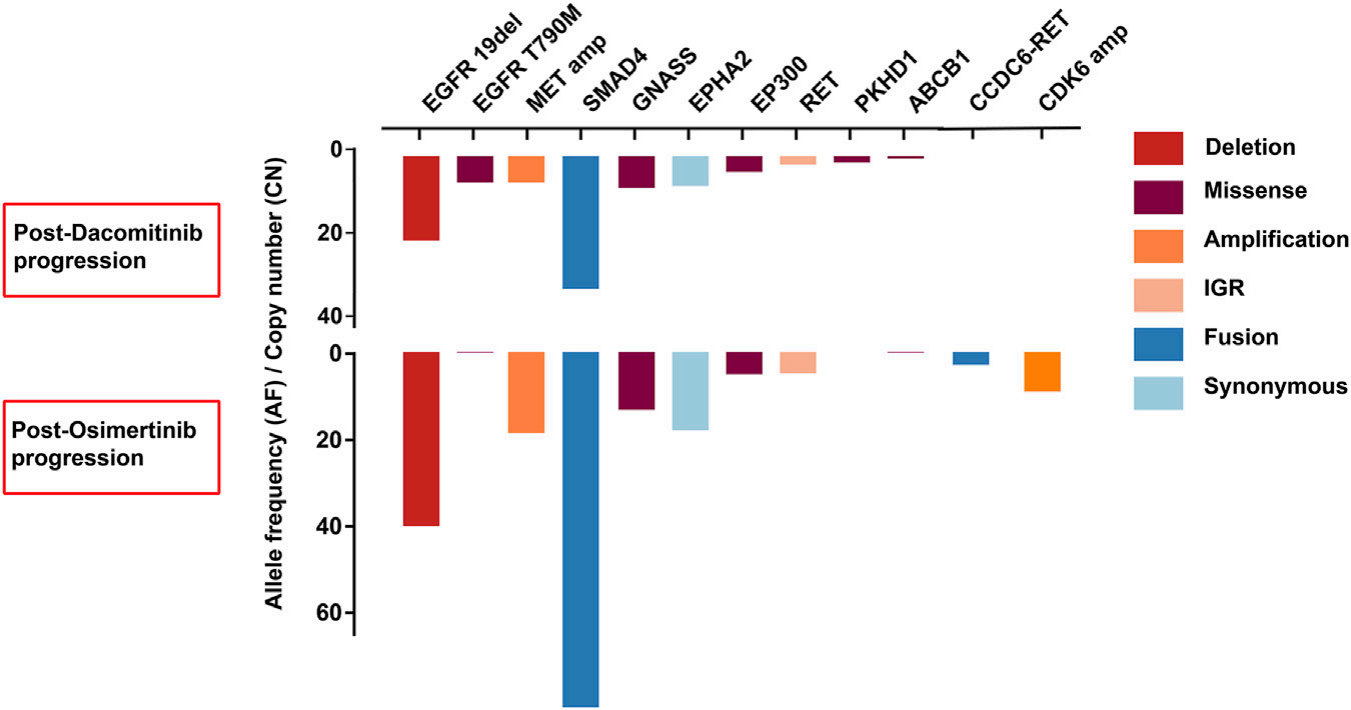
The comparison of resistance mechanisms of genetic alterations between dacomitinib progression and osimertini progression in case 2.

A 72-years woman was diagnosed with lung adenocarcinoma by percutaneous left lung biopsy in August 2019. The patient had multiple bone metastases with clinical disease stage Ⅳ. The gene testing showed an EGFR 19del by plasma-based ARMS-PCR (EGFR gene mutation assay). She started dacomitinib at 45 mg daily in August 2019. The tumor shrank with a duration of response (DOR) of 19 months (Figure 2). The patient had progression on the right supraclavicular and cervical lymph nodes. The ultrasound-guided percutaneous biopsy of the right supraclavicular lymph node showed metastatic lung adenocarcinoma. Besides, tissue- and plasma-based NGS (425 gene-panel, Geneseeq Technology Inc., Nanjing) revealed an EGFR 19del, T790M mutation in both, and MET amplification (CN:4.7) in plasma. Second-line osimertinib was administrated in May 2021. The patient’s lesion shrank in the first month of osimertinib therapy. However, the supraclavicular lymph nodes enlarged and new brain metastases appeared in September 2021 with progression-free survival (PFS) of less than 4 months. The plasma-based NGS (437 gene-panel, Geneseeq Technology Inc., Nanjing) revealed an EGFR 19del, T790M mutation, MET amplification (CN:14.2), and CCDC6-RET fusion (Supplementary Table S2, Figure 3). The patient received radiotherapy for brain and subsequent osimertinib and crizotinib combination therapy in October 2021. She showed mild gastrointestinal toxicity of nausea and vomiting to the combination. The brain metastatic lesion was controlled but the primary lung lesion progressed after a 1-month combination. The patient had a poor performance status and severe symptoms of dyspnea.

## Discussion

The mechanisms of resistance were heterogeneous and the remaining unknown mechanisms need to be discovered. The acquired secondary EGFR T790M mutation could be overcome by second-line osimertinib with a median PFS of 10.1 months in the AURA3 study (Mok et al., 2017; Westover et al., 2018). The driver gene acquired resistance, such as MET, ALK, and RET alterations, are increasingly reported (Zeng et al., 2022). It is unclear if osimertinib will exert its efficacy in patients with concurrent T790M-positive and driver gene resistance.

For the first time, we reported cases harboring concurrent alterations of EGFR T790M with ALK fusion and MET amplification after EGFR-TKIs. Both patients had a PFS of less than 4 months under osimertinib second-line therapy. The presented cases showed concurrent EGFR T790M and that driver gene resistance could not benefit from osimertinib monotherapy. Besides, the two patients developed MET amplification and RET fusions after osimertinib, which indicated that subsequent new bypass activations were possible resistance mechanisms to second-line osimertinib in patients with EGFR T790M and driver gene resistance.

Further, we conducted extensive literature reviews to identify the incidence rate and prognosis of concurrent driver gene resistance. EGFR secondary T790M, accompanying ALK, ROS1, MET, RET, HER2, KRAS, or NTRK gene alterations, were included. The incidence of T790M mutation overlapping with driver gene mutation, such as HER2-and MET amplification, was 4–8% after prior EGFR-TKIs treatment (Yu et al., 2013; Gou et al., 2016; Fu et al., 2021). Wang and coworkers reported a T790M lative allele frequency less than 20% was more likely concurrent driver gene resistance, like MET and HER2-amplification in EGFR-TKIs progression patients, who had a lower objective response rate and disease control rate to osimertinib (Wang et al., 2020). Besides, the reported cases of concurrent EGFR T790M and driver gene resistance are summarized in Table 1. The patients with T790M and KRAS G12V mutation coexistence did not respond to osimertinib therapy (Xiang et al., 2015; Fu et al., 2021). The patients harboring acquired EGFR T790M, HER2 mutation, and HER2 amplification from first-generation EGFR-TKIs did not respond to osimertinib and the combination of trastuzumab with a frustrating PFS (Meedendorp et al., 2018; Zhang et al., 2018; Ralki et al., 2019). The statistics also presented the poor efficacy of osimertinib on T790M and driver gene resistance.

**TABLE 1:** Clinicopathological characteristics of concurrent EGFR-T790M and driver gene-resistant NSCLC patients.

Case no	Age	Sex	Smoking History	Pathology	Staging	Baseline Driver Gene	First-Line Therapy	Resistance Mechanisms	Second-Line Therapy	Best Response	PFS (mo)^a^
1 (Ralki et al., 2019)	60	M	Never	ADC	IV	EGFR L858R, HER2 amp	Gefitinib	EGFR T790M, HER2 S310Y mutation, HER2 amp	Osimertinib and trastuzumab	SD	4
2 (Zhang et al., 2018)	57	M	Yes	ADC	IV	EGFR L858R	Icotinib	EGFR T790M, HER2 V777L mutation, HER2 amp	Osimertinib	PD	1
3 (Meedendorp et al., 2018)	62	M	N/A	ADC	IV	EGFR L858R	Gefitinib, afatinib	EGFR T790M, HER2 amp	Osimertinib	SD	3
4 (Xiang et al., 2015)	69	M	Yes	ADC	IV	EGFR L858R	Gefitinib	EGFR T790M, KRAS G12V	Chemotherapy	PD	2^b^
5 (Fu et al., 2021)	72	M	N/A	ADC	IV	EGFR 19del	Gefitinib	EGFR T790M, KRAS G12V	Osimertinib	PD	3
6	76	F	Yes	ADC	Ⅳ	EGFR 19del	Gefitinib	EGFR T790M, STRN-ALK, EGFR amp	Osimertinib and alectinib	SD	4
7	72	F	Never	ADC	IV	EGFR 19del	Dacomitinib	EGFR T790M, MET amp	Osimertinib	SD	3

a The PFS, of second-line therapy.

b The patients had an OS, of 2 months

AbbreviationsM, male; F, female; ADC, adenocarcinoma; N/A, not available; PR, partial response; SD, stable disease; PD, progression disease; PFS, progression-free survival; mo, months.

EGFR-TKIs combined with bypass targeted therapy showed potent antitumor activity. Osimertinib plus savolitinib showed an acceptable risk-benefit in NSCLC patients with acquired MET amplification from pretreated EGFR-TKIs in a phase Ⅰb study (Sequist et al., 2020). crizotinib combined with osimertinib also demonstrated improved PFS in a real-world study (Liu et al., 2021). Patients with acquired ALK fusions resistant to EGFR-TKIs, such as EML4-ALK and STRN-ALK, may benefit from EGFR plus ALK inhibitors (Offin et al., 2018; Zhou et al., 2019). RET fusion appearance post-osimertinib progression responded to osimertinib and pralsetinib, the selective RET-TKI (Piotrowska et al., 2018). trastuzumab emtansine (T-DM1) and trastuzumab deruxtecan were available targeted agents with activity against the HER2 alterations, however, strategies against acquired HER2 alterations await more clinical evidence (Zeng et al., 2022).

Hence, strategies to rival multiple driver gene resistance are urgently needed. Firstly, the plasma assay of EGFR T790M single-point is insufficient to identify the resistance of EGFR-TKIs, since concurrent driver gene resistance impairs osimertinib’s efficacy. Osimertinib combined with bypass TKIs is a promising treatment for concurrent EGFR T790M and driver gene resistance. The circulating tumor DNA (ctDNA) genomic profile was an effective tool to observe the resistance mechanisms (Del Re et al., 2019). ctDNA monitoring may allow the timely combination of osimertinib and bypass inhibitors. When EGFR T790M is accompanied with ALK/MET/ROS1 resistance, osimertinib and crizotinib with AEs observation could be taken into consideration since crizotinib may prevent the activation of the other two mechanisms.

## Conclusion

We firstly reported lung cancer patients harboring concurrent alterations of EGFR T790M with ALK fusion, MET amplification, and RET fusions as mechanisms of resistance after EGFR-TKIs. Both patients had poor outcomes from osimertinib. Additionally, subsequent new bypass activations were possible resistance mechanisms to second-line osimertinib. T790M accompanying driver gene resistance will be a new subtype after EGFR-TKI progression and needs effective treatment options.

## Data Availability

The original contributions presented in the study are included in the article/Supplementary Material, further inquiries can be directed to the corresponding author.
