# Supplementary material for: Acquired Concurrent EGFR T790M and Driver Gene Resistance From EGFR-TKIs Hampered Osimertinib Efficacy in Advanced Lung Adenocarcinoma: Case Reports
Source: Front Pharmacol. 2022 Apr 6;13:838247. doi: 10.3389/fphar.2022.838247 (PMC9020767; doi:10.3389/fphar.2022.838247)
Supplement: Supplementary file 1 [file DataSheet1.DOCX]

**Table S1**. Genetic alterations changes at initial and different treatment periods in Patient 1

| Time | EGFR 19del (AF) | EGFR-T790M (AF) | EGFR-amp (CN) | STRN-ALK (AF) | TP53 mutation (AF) | MET-amp (CN) |
| --- | --- | --- | --- | --- | --- | --- |
| Baseline^1^ | + | - | N/A | - | N/A | N/A |
| Gefi 12 months^2^ | 20.09% | 15.01% | 2.9 | 5.01% | 11.78% | - |
| Osimer 10 weeks^2^ | 39.17% | - | 2.8 | 12.34% | 17.87% | - |
| Osimer + Alec 3 weeks^2^ | 7.8% | - | - | - | 6.9% | - |
| Osimer + Alec 7 weeks^3^ | 18.8% | - | - | - | 19.1% | 4.2 |

Abbreviations: Gefi, Gefitinib; Osimer, Osimertinib; Alec, Alectinib; Crizo, Crizotinib; N/A, Not available; AF: Allele frequency; CN: Copy number.

^1^ Tissue-based amplification refractory mutation system polymerase chain reaction;

^2^ Plasma-based next-generation sequencing.

^3^ Right iliopsoas -based next-generation sequencing

**Table S2** The distribution of genetic alterations after Dacomitinib and Osimertini resistance in patient 2

| Genetic alterations | Alterations types | Dacomitinib progression  (AF/ CN) | Osimertinib progression (AF/ CN) |
| --- | --- | --- | --- |
| EGFR 19del | Deletion | 15.2% | 30.5% |
| EGFR T790M | Missense | 4.7% | 0.1% |
| MET amp | Amplification | 4.7 | 14.2 |
| CCDC6-RET | Fusion | - | 2.2% |
| CDK6 amp | Amplification | - | 6.9 |
| SMAD4-DTNB | Fusion | 24.0% | 62.2% |
| GNASS R232C | Missense | 5.7% | 10.1% |
| EPHA2 | Synonymous | 5.3% | 13.7% |
| EP300 P1039L | Missense | 2.8% | 3.8% |
| RET | IGR | 1.5% | 3.7% |
| PKHD1 L107M | Missense | 1.1% | - |
| ABCB1 Q143H | Missense | 0.4% | 0.1% |

Abbreviations: Allele frequency; CN: Copy number.
